# Safety, pharmacokinetic, and pharmacodynamic characteristics of SK-08, a soluble guanylate cyclase activator, after oral administration in healthy participants: a randomized phase 1 trial

**DOI:** 10.1186/s12882-026-05138-y

**Published:** 2026-07-09

**Authors:** Jian Liu, Ying Du, Hong Zhou, Hanghuan Jia, You Zhai, Jie Ruan, Caixia Yan, Nana Xu, Yijing Xin, Liya Zheng, Yingying Song, Danna Jiang, Jing Yang

**Affiliations:** 1 Department of Clinical Pharmacy, The First Affiliated Hospital, Zhejiang Provincial Key Laboratory for Drug Evaluation and Clinical Research, Zhejiang University School of Medicine, Hangzhou, China; 2 Consun Pharmaceutical Group Limited, Guangzhou, Guangdong 510006 China

**Keywords:** Soluble guanylate cyclase activator, Phase 1, Food effect, Pharmacodynamic, Pharmacokinetic, Safety

## Abstract

**Background:**

Uncoupling of the nitric oxide (NO)-soluble guanylate cyclase (sGC)-cyclic guanosine monophosphate (cGMP) axis and impaired cGMP synthesis are hallmarks of chronic kidney disease (CKD). While sGC has been a target of therapeutic interest for CKD, clinical progress has been limited.

**Methods:**

In this single center, phase 1 study (NCT07021157), the pharmacokinetics (PK), pharmacodynamics (PD), and safety of SK-08, a novel, oral sGC activator, were evaluated. Healthy participants were randomized to SK-08 or placebo in the single-ascending-dose phase; in the food-effect phase, SK-08 10 mg was administered under fasted and fed conditions. cGMP was the key PD biomarker, and exposure-response analyses were performed.

**Results:**

Between 18th March 2025–17th June 2025, 16 participants were randomized and received SK-08 5 mg (*n* = 3), 7.5 mg (*n* = 3), or 10 mg (*n* = 6), or placebo (*n* = 4). Adverse events (AEs) were mild/moderate and resolved spontaneously, those with an incidence > 10% were hypotension (4/12, 33.3%) and dizziness (2/12, 16.7%). A higher C_max_ was associated with an increased incidence of adverse drug reactions (ADRs) across all SK-08 doses. Under fasted conditions, the absorption and time to peak plasma concentration of SK-08 was fairly rapid (median T_max_ 2.5–5.0 h). Administration after a high-fat meal prolonged the median T_max_ by 0.7 h; C_max_ and AUC were decreased by ~ 10%. Mean cGMP maximum percentage change (E_max_) increased dose dependently, from 5.99% at 5 mg to 27.19% at 10 mg, with a median time to E_max of_ 2.75–6.00 h.

**Conclusions:**

SK-08 was well tolerated in healthy participants and induced dose-related sGC activation. A potential association between C_max_ levels and the incidence of ADRs, and no clinically meaningful impact of food, was observed. These findings guide the optimization of a controlled-release formulation to maximize the therapeutic potential of SK-08.

**Trial registration:**

Chinadrugtrials.org.cn (CTR20250337). Registered 20,250,126 Clinicaltrials.gov (NCT07021157). Registered 20,250,512.

**Supplementary Information:**

The online version contains supplementary material available at https://doi.org/10.1186/s12882-026-05138-y.

## Background

The global mortality from chronic kidney disease (CKD) increased by 41.5% from 1990 to 2017 [1], and a 2021 estimate reported 673.7 million cases of CKD globally [2]. CKD has a particularly high prevalence in Asia; up to 434.4 million adults (95% confidence interval [CI] 350.2–519.7) are estimated to have CKD in Eastern, Southern and South-Eastern Asia [3]. Patients with CKD have an elevated cardiovascular risk [4, 5], and cardiovascular mortality accounts for up to 50% of all deaths in patients with advanced CKD. Despite the efficacy of first-line treatments such as angiotensin-converting enzyme inhibitors (ACEIs) and sodium-glucose cotransporter-2 inhibitors (SGLT2is) in slowing CKD progression and reducing proteinuria [6], a high risk of advancement to end-stage renal and cardiovascular disease persists [7]. As a result, novel therapies that can reduce the cardiorenal risk associated with CKD are required.

Soluble guanylate cyclase (sGC), a heterodimeric complex composed of two subunits (α and β), is a key enzyme in the nitric oxide (NO)-cyclic guanosine 3’,5’-monophosphate (cGMP) signaling cascade (NO-sGC-cGMP pathway), which is pivotal in vascular homeostasis, and exerts renal protective effects through systemic action on the cardiovascular system [7, 8]. Impaired NO signaling is regarded as a hallmark of cardiovascular diseases as it results in endothelial dysfunction [8]. Importantly, oxidative damage and inflammation that cause the uncoupling of NO-cGMP signaling and low cGMP synthesis lead to kidney damage and the progression of CKD [7]. Therefore, sGC activators, which increase cGMP concentration, may provide an effective treatment for renal diseases.

A number of pre-clinical studies support a renal protective effect of sGC modulators; demonstrating improved renal blood flow as well as reduced interstitial inflammation, fibrosis, glomerulosclerosis, and albuminuria [8–11]. Vericiguat isa marketed sGC modulator indicated for heart failure which established the significant cardioprotective effects of this class of drugs. In addition, clinical data for the sGC activator runcaciguat demonstrate significant reductions in urine albumin-to-creatinine ratio in patients with diabetic kidney disease [12]. However, despite the promising evidence, the clinical development of sGC activators for CKD treatment has faced major hurdles including inconsistent responses in comorbid patients and complex dosing regimens requiring multiple doses and titration, which reduce long-term adherence.

SK-08 is a novel sGC activator being investigated for the treatment of CKD. Preclinical pharmacodynamics (PD) studies indicate that SK-08 may provide a safe and effective treatment option for patients with CKD. For example, in DOCA-salt hypertensive rats, SK-08 demonstrated a dose-dependent improvement in key parameters including reduced blood pressure, lowered urinary protein and serum urea nitrogen, and increased glomerular filtration rate corrected for kidney weight (eGFR/KW), with the efficacy at 6 mg/kg matching that of runcaciguat 8 mg/kg. Consistent with these observations, SK-08 exhibited superior potency to runcaciguat in an LNCaP cell-based sGC activation assay, showing approximately 3-fold greater potency (EC_50_: 21.19 vs. 75.97 nM; MEC: 6.93 vs. 27.11 nM) (data on file).

Here, we report the results from a randomized, placebo-controlled phase 1 study evaluating the safety, tolerability, pharmacokinetic (PK), and PD of single ascending oral doses of SK-08 in healthy participants. The trial was designed to support formulation optimization while providing proof-of-mechanism of SK-08 and confirming that a flat, sustained PK profile is essential for a balanced safety-efficacy profile of sGC activators. Collectively, these results will guide the clinical advancement of SK-08 and help overcome the recognized challenges of this drug class.

## Methods

The protocol and all relevant documents were approved by the local Ethics Review Board (Clinical Trial Ethics Committee of The First Affiliated Hospital, College of Medicine, Zhejiang University [2024 approval number 827]). The trial was conducted in accordance with the Chinese Guidelines for Good Clinical Practice, applicable local laws and regulations, and the principles outlined in the Declaration of Helsinki and its subsequent revisions. All participants provided written, informed consent before participation. The study was registered at Chinadrugtrials.org.cn (CTR20250337) prior to participant enrolment, on 26th January 2025, and at ClinicalTrials.gov (NCT07021157) on May 12, 2025.

### Study design

This was a single-center, randomized, double-blind, placebo-controlled, phase 1 dose escalation study including a nested, two-period, fasted-fed sequence food-effect evaluation. The study was conducted at the Phase I Clinical Trial Center of The First Affiliated Hospital of Zhejiang University School of Medicine, Hangzhou, China.

Seven dose cohorts were prespecified, starting at 5 mg. Subsequent cohorts were planned with dose increments of 2.5–7.5 mg, with each increase not exceeding 50% of the previous dose, up to a maximum of 30 mg. For cohorts 1–2, four participants were planned to be enrolled (three to SK-08, one to placebo), while for cohorts 3–7, eight participants were planned to be enrolled (six to SK-08, two to placebo).

## Dose level justification

The 5 mg starting dose is 50% of the human equivalent dose converted from the effective dose observed in rat hypertension and diabetic nephropathy models. It is also 20 times lower than the human equivalent dose (HED) corresponding to the no-observed-adverse-effect level (NOAEL) in a 28-day Good Laboratory Practice (GLP) long-term toxicity study, in which SK-08 was administered to rats via repeated oral gavage once daily. Subsequent dose levels were determined in dose escalation meetings based on the safety, PK, and PD results of previous cohorts, with the maximum dose increase < 50% and decreasing with increasing dose. The maximum proposed dose was 30 mg; referring to the “Guidance on Nonclinical Safety Studies for the Conduct of Clinical Trials and Marketing of Pharmaceuticals (M3(R2))”, this dose is much lower than 50% of the NOAEL exposure in the long-term preclinical toxicity study.

## Participants

Healthy male or female volunteers 18–45 years of age with a body weight ≥ 50 kg for males or ≥ 45 kg for females, and a body mass index (BMI) between 19.0 and 26.0 kg/m^2^ were eligible. Participants were required to be in good health based on medical history, physical examination, vital signs, 12-lead electrocardiogram (ECG), abdominal ultrasound, and clinical laboratory tests.

Key exclusion criteria included: a clinically significant disorder/condition; a 12-lead ECG demonstrating heart rate < 40 beats per minute (bpm) or average QT interval corrected using Fridericia’s formula (QTcF) > 450 ms for males or > 470 ms for females; alanine aminotransferase, aspartate aminotransferase, or creatine > 1.25 x the upper limit of normal; a history or presence of hypotension or hypovolemia; and a history or presence of syncope or frequent dizziness (≥ 2 episodes in the past 6 months). A full list of exclusion criteria is provided in the supplemental materials.

## Procedures

SK-08 (Consun Pharmaceutical [Inner Mongolia] Co., Ltd.) was supplied as film-coated tablets. Participants were randomized via a centralized, computer-generated schedule to receive SK-08 or placebo in a 3:1 ratio.

In the dose-escalation phase under fasting conditions (≥ 10 h before dosing), participants were confined to the clinic from Day − 2. A single oral dose of SK-08 was administered on Day 1, followed by a standard meal 4 h post-dose (see Supplementary Appendix for meal composition). Participants in the 5 and 7.5 mg cohorts were discharged after a safety check on Day 5 and returned for a follow-up visit on Day 12.

The 10 mg cohort additionally underwent a food-effect assessment. This segment nested a two-period, sequential design: following the initial fasted dose and a 7-day washout period, participants received a second dose 30 min after starting a high-fat meal (900–1000 kcal). All participants were discharged following safety reviews on Day 12 and returned for a final follow-up on Day 19.

The concomitant use of other medications, dietary supplements, and non-drug therapies was prohibited during the study.

## Measurements and outcomes

The primary endpoint was safety, assessed through recording adverse events (AEs), vital signs, 12-lead ECG (QTcF), and laboratory tests, with the measurements made in triplicate. AEs were coded using MedDRA and graded per the Common Terminology Criteria for Adverse Events (CTCAE) v5.0 and the Chinese phase 1 healthy volunteer AE guidelines [13].

Secondary endpoints were estimation of PK and PD parameters. Blood samples for PK analysis were scheduled to be collected within 1.00 h prior to dosing and at 0.50, 1.00, 1.50, 2.00, 2.25, 2.50, 2.75, 3.00, 3.50, 4.00, 5.00, 6.00, 8.00, 12.00, 24.00, 48.00, 72.00, and 96.00 h post-dose. Approximately 4 mL of venous blood was collected at each timepoint. Blood samples for PD assessment of cGMP were scheduled to be collected within 1.0 h prior to dosing and at 0.50, 1.00, 1.50, 2.00, 2.50, 3.00, 4.00, 5.00, 6.00, 8.00, and 12.00 h post-dose.

Heart rate and blood pressure were measured within 1.0 h prior to dosing and at approximately 1.0 h ± 20 min, 2.0 ± 0.5 h, 4.0 ± 0.5 h, 6.0 ± 0.5 h, and 12.0 ± 0.5 h post-dose. When these assessments coincided with routine vital sign monitoring, blood pressure values from the vital sign schedule could be used without repeated measurement. Heart rate was determined as the mean of three measurements taken within 5 min of the QT interval assessment at each timepoint. For subsequent dose cohorts, the number and timing of assessments could be adjusted based on emerging trial results.

### PK and PD methods

For PK sample collection, approximately 4 mL of venous blood was collected at each time point into an EDTA-K_2_ anticoagulant PK vacuum blood collection tube. The tube was inverted 5–10 times to ensure complete mixing. Blood samples were centrifuged at 1700 g (+/-100 g), 4 °C (2–8 °C) for 10 min within 1 h of collection. After centrifugation, approximately 1.0 mL of the upper layer (plasma) was drawn from the PK collection tube into a testing tube, and the remaining plasma was aliquoted into a backup tube. The plasma samples in the testing and backup tubes were stored at -80 °C (-60 to -90 °C) until analysis.

For PD analysis, 3 mL venous blood at each sampling time point was collected into a vacuum blood collection tube containing the EDTA-K_2_ anticoagulant and inverted 5–10 times. Blood samples were centrifuged at 1700 +/- 100 g, 4 °C (2–8 °C) for 10 min, within 1 h of collection. Immediately after centrifugation, 0.8 mL of plasma was aliquoted into a test aliquot storage tube containing 0.08 mL of stabilizer (2% formic acid aqueous solution) and a vortex mixer was used to mix thoroughly (for at least 5 s). The plasma samples were stored at -80 °C (-60 to -90 °C) within 2 h after centrifugation.

Concentration of SK-08 and cGMP was estimated using a validated High-Performance Liquid Chromatography coupled with Tandem Mass Spectrometry (HPLC–MS/MS) method. For PK analysis, compounds were detected using an API-5500 in positive-ion mode, and carbamazepine was used as the internal standard. HPLC-MS/MS was conducted using a Triple Quad™ 6500+ (AB SCIEX) mass spectrometer with a turbo ion spray source, coupled with a LC-40 AD XR UHPLC (Shimadzu). Human plasma samples were pretreated via liquid-liquid extraction using methyl tert-butyl ether as the extraction solvent. For each analytical batch, the linearity of the calibration curve and the suitability of quality control (QC) samples were verified. Calibration curves were constructed over a concentration range of 0.500–500 ng/mL in plasma, with correlation coefficients (r) > 0.9950 for all batches. The accuracy of low-, medium-, and high-concentration QC samples was assessed to confirm the reliability of the analysis. Accuracy bias was within ± 15% (± 20% for the lower limit of quantification [LLOQ] QC), and precision (%RSD) was ≤ 15% (≤ 20% for LLOQ QC). The concentration of SK-08 in plasma was determined by the ratio of the analyte peak area to the internal standard peak area obtained from LC-MS/MS analysis.

Sodium citrate plasma cGMP samples were processed by protein precipitation extraction and separated by reverse-phase chromatography with a mobile phase gradient of 0.1% acetic acid in water and 50:50 acetonitrile: methanol. Analytes were detected using an API-5500 with negative-ion electrospray ionization. Human plasma samples were pretreated by protein precipitation, with the precipitant consisting of acetonitrile–water (80:20, v: v) containing 0.1% formic acid. The calibration curves were established over the concentration range of 0.100–40 ng/mL in human plasma, and the correlation coefficient (r) of the regression equation exceeded 0.9910 across all analytical runs. The validation items for cGMP analysis, including calibration curve linearity, applicability of quality control samples, accuracy and precision of QC samples, as well as the concentration calculation method, were entirely consistent with those adopted in PK analysis.

The validation of the bioanalytical methods for PK samples was conducted in compliance with the ICH M10 (Bioanalytical Method Validation and Study Sample Analysis guideline), and the principles outlined in the Chinese Pharmacopoeia 2020, Part IV, Appendix 9012 (Guideline for Validation of Quantitative Bioanalytical Methods for Biological Samples, published by the Chinese Pharmacopoeia Commission). cGMP was measured as a key PD biomarker using a fit-for-purpose LC-MS/MS method.

### Statistical analysis

No formal sample size calculation was performed. Enrollment of up to 48 participants was planned, sufficient to provide safety data and preliminary PK and PD profile. Safety was summarized descriptively.

PK parameters were estimated using a noncompartmental analysis (WinNonlin, Certara). Parameters estimated included maximum plasma concentration (C_max_), time to maximum plasma concentration (T_max_ ), area under the concentration time curve (AUC) from 0 to the time of the last quantifiable concentration (AUC_0–t_), AUC from time 0 to infinity (AUC_0–∞_), the percentage of AUC due to extrapolation beyond the last measurable concentration (AUC__%Extrap_), elimination half-life (T_1/2_), λ_z_, apparent clearance (CL/F), and apparent volume of distribution adjusted for bioavailability (V_z_/F). PD endpoints included changes in systolic, diastolic, and mean arterial pressure, heart rate, and plasma cGMP. Analyses included baseline-corrected, placebo-corrected, and percentage changes from baseline.

A power model expressed as Y = Exp(α) (Dose)^β Exp(ε) (or equivalently the linear form ( ln(Y)=α + β ⋅ ln(Dose)+ε) was used for the PK linearity analysis, where α incorporates fixed period effects and random subject effects, β indicates perfect dose proportionality, ε represents the error term [14], and dose proportionality was determined if the (1–α)×100% confidence interval of the slope β lied within the pre-specified equivalence limits [15]. The θ was a prespecified limit; typically with θ_L_ = 0.8 and θ_H_ = 1.25 for AUC, and θ_L_ = 0.7, θ_H_ = 1.43 for C_max_. The r was 2 as the ratio of the highest: lowest dose.

$$\:\mathrm{Equivalent\:limits:\:1+}\frac{\mathrm{ln}\left({\theta}_{\mathrm{L}}\right)}{\mathrm{ln}\left(\mathrm{{\gamma}}\right)}\text<{\beta}<1+\frac{\mathrm{ln}\left({\theta}_{\mathrm{H}}\right)}{\mathrm{ln}\left(\mathrm{{\gamma}}\right)}$$

For the food-effect study, log-transformed C_max_, AUC_0–t_, and AUC_0–∞_ were analyzed using a mixed-effects model with fed versus fasted as a fixed effect and subject as a random effect. Geometric mean ratios (fed/fasted) and associated 90% confidence intervals (CIs) were calculated. Food was considered to have no significant effect if the 90% CIs fell within 80–125%. Statistical analyses were performed using Phoenix WinNonlin (version 8.3 or higher) and SAS (version 9.4 or higher).

## Results

### Participants

**Fig. 1:**
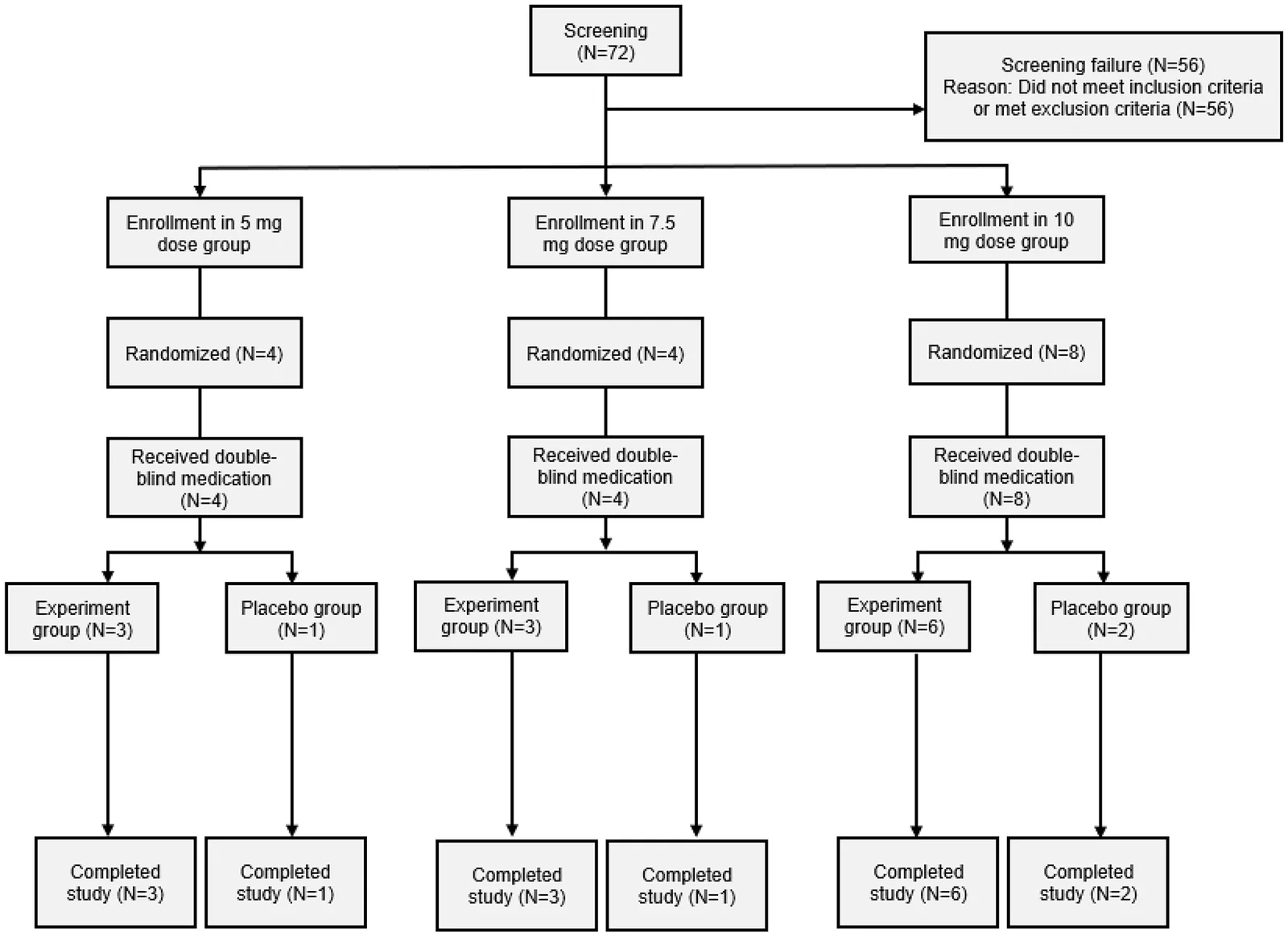
Participant disposition flow chart. Note: Participants enrolled in the 10 mg dosage group received SK-08 in both fasted and fed conditions, with a 7-day washout period

Between 18th March 2025 and 17th June 2025, a total of 16 participants were enrolled and completed drug administration as scheduled in the protocol; 12 received SK-08, and 4 received placebo. Four participants each were enrolled in the 5 and 7.5 mg groups, and eight participants in the 10 mg group (Fig. 1). Dose escalation was terminated after the third cohort in consideration of participant safety, as adequate data had been generated to support formulation modification; the human PK characteristics and the preliminary exposure-response relationship (for both cGMP levels and safety profile) were established from data collected in the first three cohorts and ADRs occurred in 83.3% of patients in the 10 mg cohort. All eight participants in the 10 mg group completed fasted administration as planned and received fed administration after a 7-day washout period. Participant demographics and baseline characteristics were generally balanced across the dosage groups, with a mean (standard deviation [SD]) age of 31.4 (5.38) years (Table 1). Height, weight, and BMI were similar across the dose groups. However, it should be noted that there was a sex imbalance between the dose groups, with no males in the 7.5 mg group and a high proportion of males in the placebo group.

**Table 1:** Participant demographics and characteristics

Variable	Statistic	Experimental group			Placebo	Total
		5 mg (*N* = 3)	7.5 mg (*N* = 3)	10 mg (*N* = 6)	(*N* = 4)	(*N* = 16)
**Age (years)**	Mean (SD)	28.3 (5.0)	34.0 (5.6)	35.2 (3.4)	26.0 (2.2)	31.4 (5.4)
**Sex** **n (%)**	Male	2 (66.7)	0	4 (66.7)	3 (75.0)	9 (56.3)
	Female	1 (33.3)	3 (100.0)	2 (33.3)	1 (25.0)	7 (43.8)
**BMI (kg/m** ^**2**^ **)**	Mean (SD)	25.2 (0.53)	23.60 (1.49)	22.6 (1.67)	23.9 (2.42)	23.6 (1.83)

BMI, Body mass index; SD, Standard deviation

### Safety

Overall, observed AEs were mild to moderate in severity and resolved spontaneously. AEs with an incidence > 10% were hypotension (33.3%) and dizziness (16.7%), and no participants had a QTcF > 450 ms or ΔQTcF > 60 ms. Under fasted conditions, the overall incidence of adverse drug reactions (ADRs) for SK-08 was 58.3% (7/12), compared to AE rate of 75.0% (3/4) with placebo. A dose-related increase of ADRs was observed in the SK-08 fasted cohort, with incidences of 33.3% (1/3) at 5 mg, 33.3% (1/3) at 7.5 mg, and 83.3% (5/6) at 10 mg. The incidence of ADRs at the 10 mg dose was lower under fed (50.0%) versus fasted conditions. Drug-related hypotension was reported in one participant at 7.5 mg (33.3%) and three at 10 mg (50.0%) (Table 2). All cases of hypotension occurred exclusively in the fasted period. No deaths or serious AEs were observed.

**Table 2:** Summary of adverse events by group

Adverse event	5 mg, fasted (*N* = 3)	7.5 mg, fasted (*N* = 3)	10 mg, fasted (*N* = 6)	Overall (*N* = 12)	10 mg, fed (*N* = 6)	Placebo (*N* = 4)
	2 (66.7)	2 (66.7)	6 (100)	10 (83.3)	3 (50.0)	3 (75.0)
**Adverse drug reaction**	1 (33.3)	1 (33.3)	5 (83.3)	7 (58.3)	3 (50.0)	2 (50.0)
**Drug-related hypotension**	0	1 (33.3)	3 (50.0)	/	0	/
**SAEs**	0	0	0	0	0	0
**AEs leading to treatment discontinuation**	0	0	0	0	0	0
**Deaths**	0	0	0	0	0	0

AE, Adverse event; SAE, Serious adverse event

### Pharmacokinetics

**Fig. 2:**
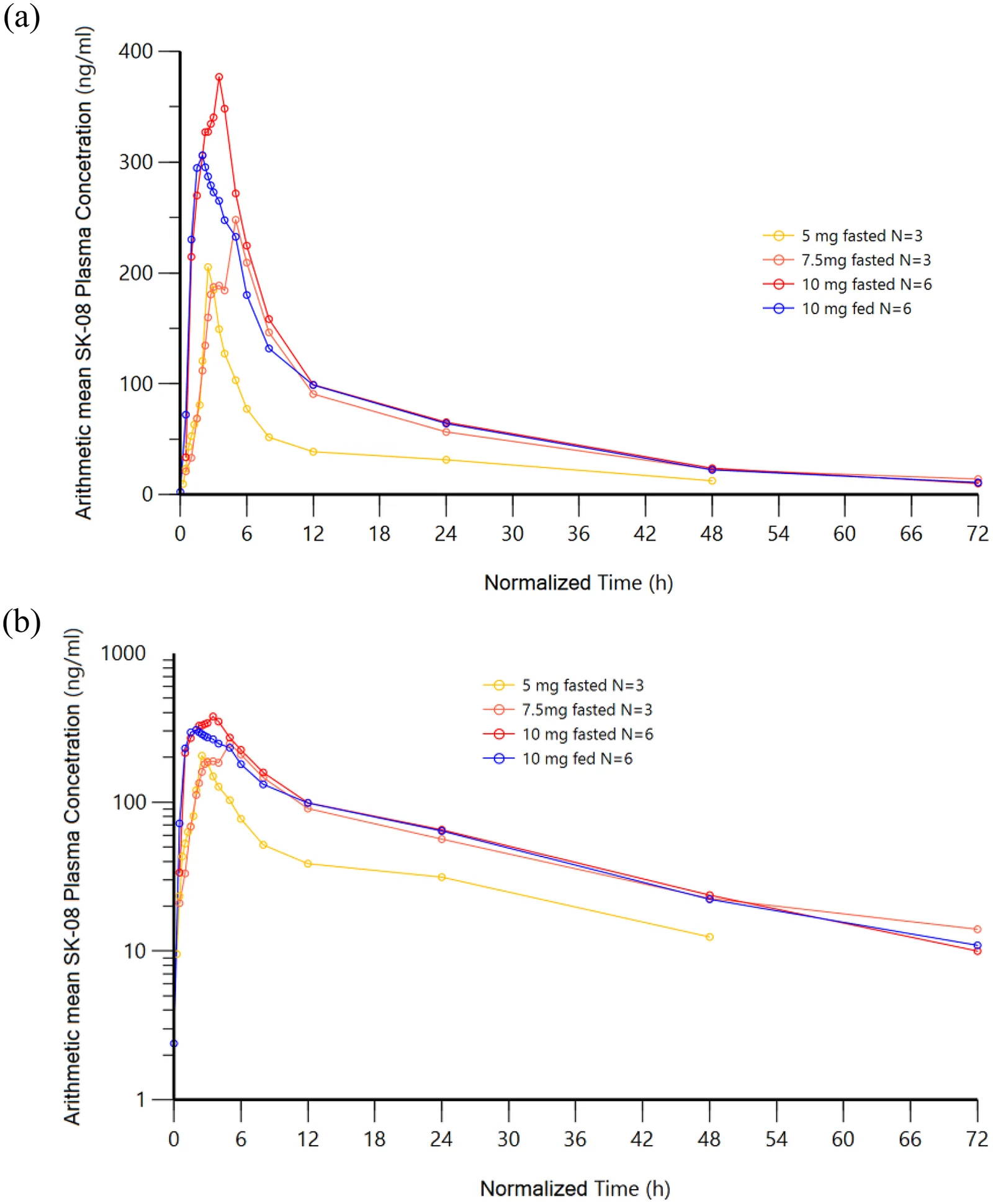
Arithmetic mean plasma concentration-time profiles of SK-08. Note: (**a**) linear scale and (**b**) semi-logarithmic scale

Following a single oral administration under fasted conditions, the absorption and time to peak plasma concentration of SK-08 was fairly rapid across all dose groups, with a median T_max_ of 2.5–5.0 h (except for individual participants in the 7.5 mg group, whose T_max_ was as late as 6 h) (Table 3). The plasma exposure (C_max_, AUC_0−t_) increased with dose, and the inter-individual variability in exposure was moderate (< 40%). The geometric mean values for C_max_ and AUC_0−t_ ranged between 202.1 and 430.3 ng/mL and 1813.1–4924.6 h*ng/mL, respectively (Table 3; Fig. 2).

**Table 3:** Summary of the pharmacokinetics of SK-08

PK parameter (unit)	GeoMean (GeoCV%)				
	5 mg fasted (*N* = 3)	7.5 mg fasted (*N* = 3)	10 mg fasted (*N* = 6)	10 mg fed (*N* = 6)	
**C**_**max**_ **(ng/mL)**	202.1 (21.8%)	273.7 (16.9%)	430.3 (19.0%)	375.2 (25.0%)	
**AUC**_**0−t**_ **(h.ng/mL)**	1813.1 (36.2%)	4252.0 (3.8%)	4924.6 (31.0%)	4462.8 (39.9%)	
**AUC**_**0−∞**_ **(h.ng/mL)**	1909.8	4305.2; 4244.2	5097.9(32.7%)	4596.9 (40.5%)	
**T**_**max**_ **(h)**	2.5 (2.5, 2.5)	5.0 (3.5, 6.0)	2.6 (1.0, 3.5)	1.9 (1.5, 5.0)	
**t**_**1/2**_ **(h)**	12.9	12.5; 21.8	17.1 (48.2%)	18.5 (37.3%)	
**CL/F (L/h)**	2.6	1.7; 1.8	2.0 (32.7%)	2.1 (40.6%)	
**V**_**z**_ **/F (L)**	48.6	31.4; 55.6	48.5 (40.0%)	58.2 (46.9%)	

Note 1: T_max_: Median (min, max); note 2: When *n* ≤ 2, individual values are shown

AUC_0−t_, area under the plasma concentration time curve from dosing to last measurable concentration; AUC_**0−∞**_, area under the plasma concentration time curve from zero to infinity; C_max_, peak plasma concentration; CL/F, apparent oral clearance; CV, coefficient of variation; PK, pharmacokinetic; SD, standard deviation; T_1/2_, elimination half-life; T_max_, time to C_max_, V_Z_ /F, apparent volume of distribution

The elimination phase showed a multi-phase and rapid elimination pattern, with a fast initial phase followed by a slow phase. At 24 h after administration, the plasma drug concentration decreased to 1/10 of C_max_, and the t_1/2_ was relatively long (~ 20 h) without significant dose variability (Fig. 2). Under fasted conditions, the geometric mean values for apparent clearance (CL/F) were 2.62, 1.74, and 1.96 for 5, 7.5, and 10 mg, respectively. A low percentage of AUC_0−∞_ was calculated by extrapolating from last measurable concentration to infinity (AUC__%Extrap_), suggesting that the sampling time was adequate and the elimination phase was well characterized (Table 2). No statistically significant gender differences in PK parameters (lnC_max_, lnAUC_0−t,_ and lnAUC_0−∞_) were observed in the 10 mg group under fasted conditions (t-test or Wilcoxon rank-sum test, *P* > 0.05).

Among six participants who received oral SK-08 after a high-fat meal, median T_max_ was slightly advanced, by 0.7 h, compared with the fasted state. The maximum delay of T_max_ was from 3.5 h (fasted) to 5.0 h (fed), and C_max_ and AUC were decreased by approximately 10% for fed versus fasted administration (Tables 3 and 4).

**Table 4:** Effect of food on PK parameters of SK-08

PK parameter (unit)	*N*		Geometric mean and ratio					
	Fasted	Fed	Fasted	Fed	(Fed/Fasted)%	%CV	90% CI	Power%
**C**_**max**_ **(ng/mL)**	6	6	430.312	375.199	87.2	21.994	67.71 ~ 112.28	7.31
**AUC**_**0−t**_ **(h.ng/mL)**	6	6	4924.625	4462.797	90.6	8.088	82.50 ~ 99.55	80.01
**AUC**_**0−∞**_ **(h.ng/mL)**	6	6	5097.858	4596.894	90.2	8.591	81.61 ~ 99.63	72.74

AUC_0−t_, area under the plasma concentration time curve from dosing to last measurable concentration; AUC_**0−∞**_, area under the plasma concentration time curve from zero to infinity; C_max_, peak plasma concentration

**Fig. 3:**
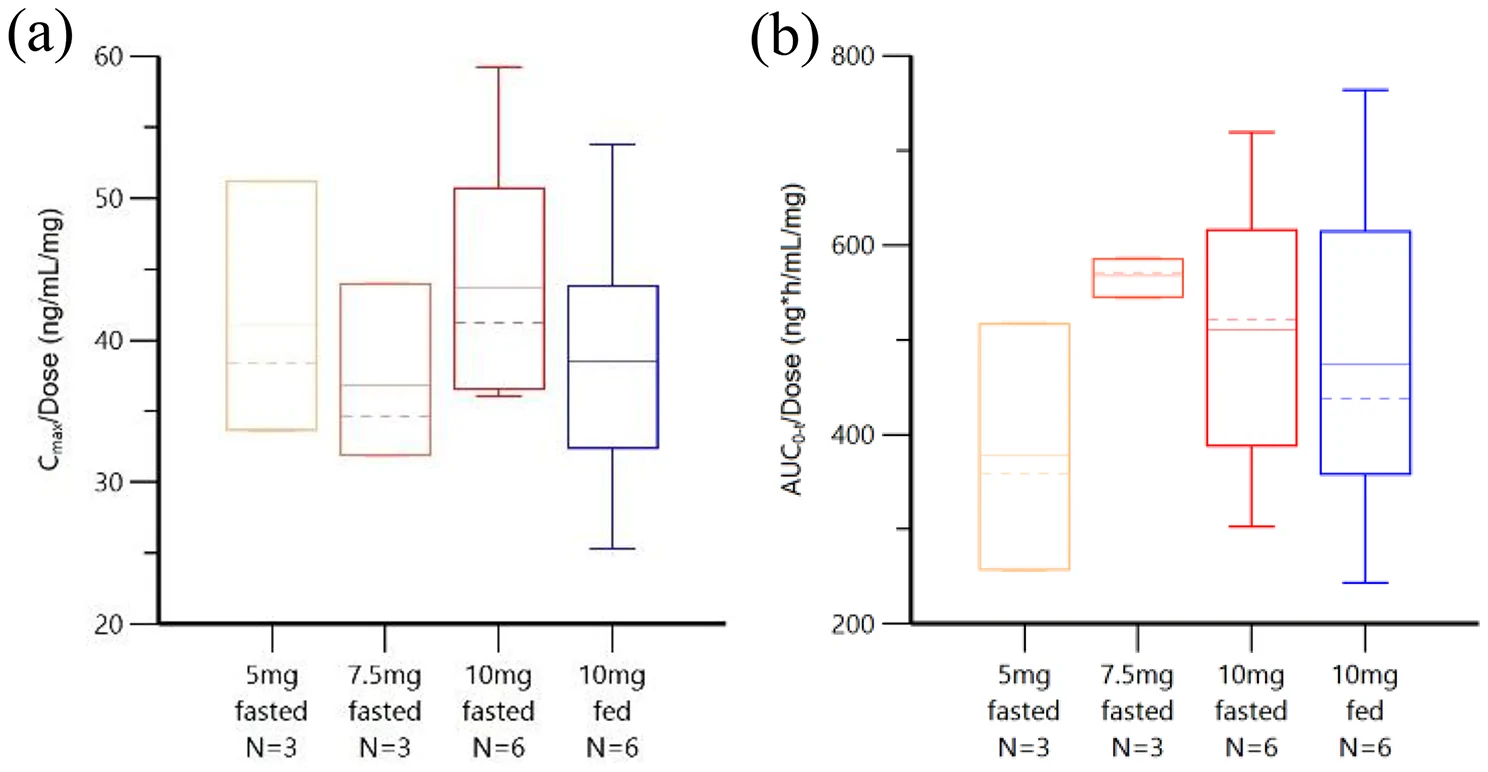
Box-whiskers-plots of dose-normalized SK-08 exposure. Note: (**a**) Cmax/dose and (**b**) AUC0-t/dose. Box plot details: median (solid line), arithmetic mean (dashed line); IQR (Q1–Q3, middle 50% of data); 1.5×IQR whiskers.

Following a single administration of SK-08 at doses of 5, 7.5, and 10 mg, a dose-proportional exposure relationship could not be determined. A preliminary evaluation of dose proportionality was made using the power model, and the resulting 90% confidence intervals (90% CIs) were 0.75–1.46 for C_max_ and 0.86–1.95 for AUC_0−t_, respectively (Table 5). The 90% CIs of AUC_0−t_ did not fall within the equivalent limit of 0.6781 ~ 1.3219, indicating further investigation within a wider dose range is required. An exposure-normalized box plot showed that the increased amplitude of C_max_ and AUC_0−t_ was greater than the dose ratio (Fig. 3). However, due to the narrow dose range and small participant number, further investigation is required to confirm these results.

**Table 5:** Dose proportionality for SK-08 from 5 to 10 mg fasted using the power model

PK parameter	Estimate (β)	*P*-value	90% CI	Equivalent limits
**ln(C**_**max**_ **)**	1.11	0.0002	0.7544 ~ 1.4624	0.4854 ~ 1.5160
**ln(AUC**_**0−t**_ **)**	1.41	0.0008	0.8636 ~ 1.9490	0.6781 ~ 1.3219
**ln(AUC**_**0−∞**_ **)**	1.27	0.0196	0.4717 ~ 2.0706	0.6781 ~ 1.3219

ln(C_max_), natural logarithm of C_max_; ln(AUC_0−t_), natural logarithm of AUC_0−t_; ln(AUC_0−∞),_ natural logarithm of AUC_0−∞_

### Pharmacodynamics

**Fig. 4:**
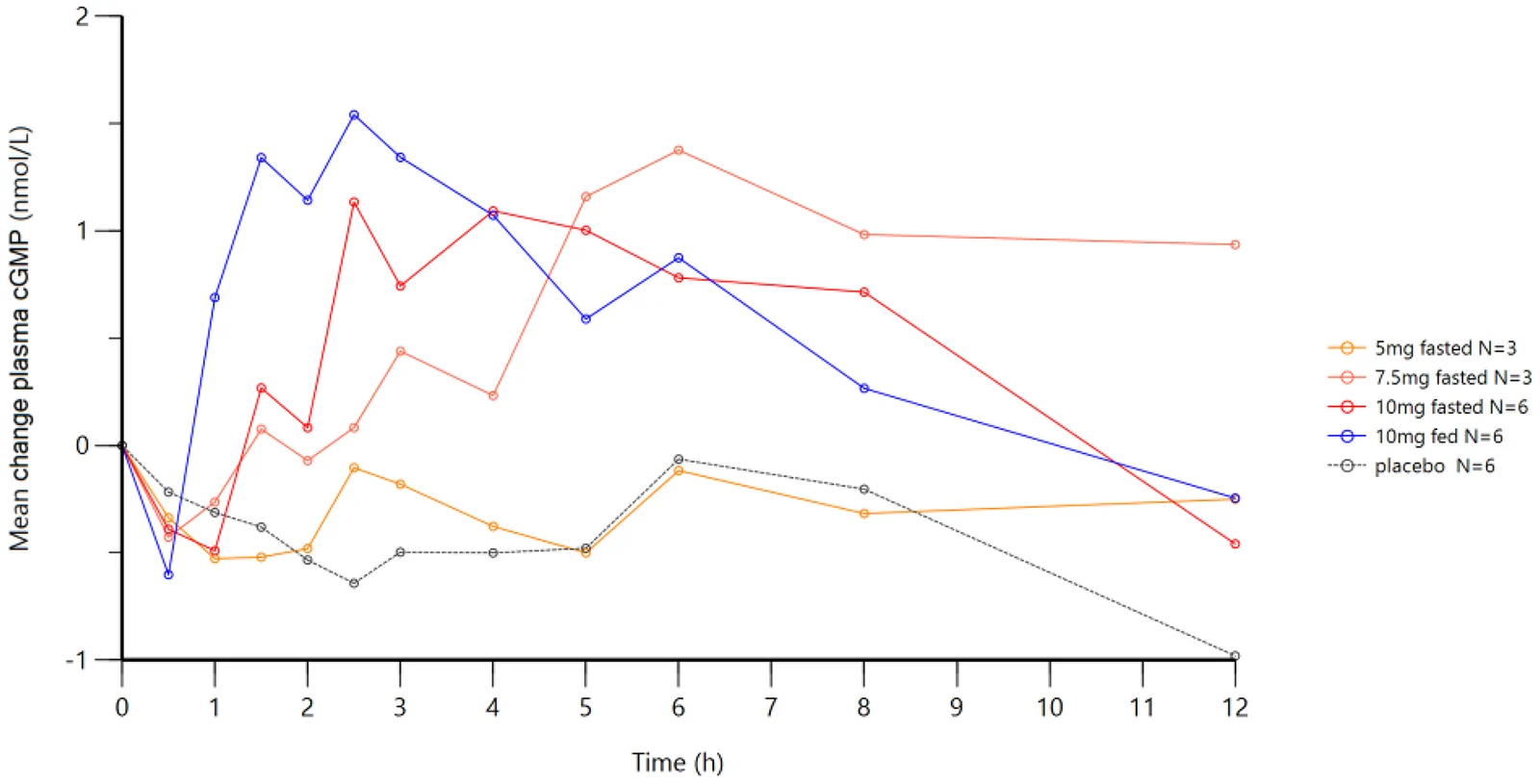
Arithmetic mean cGMP concentration change from baseline after dosing

Following administration of sGC activators, intracellular cGMP alterations constitute a direct indicator for sGC activation; consequently, increased cGMP levels elicit vasodilation and subsequent hypotension. Dose-related increases from baseline in mean plasma cGMP were observed following a single dose of SK-08. Maximal increases from baseline typically coincided with the T_max_ of SK-08 plasma concentration, and mean values remained above placebo for up to 8 h post dose (Fig. 4). The changes in cGMP concentration after double correction for baseline and placebo showed the same trend.

Since cGMP is an endogenous substance in the human body and exhibits circadian rhythmic changes, the maximum percentage change (E_max_) from baseline within 12 h post-dose was used to assess the effect of SK-08. The mean E_max_ values were 5.99% in the 5 mg group, 31.21% in the 7.5 mg group, and 27.19% in the 10 mg group under fasted conditions, and 34.94% in the 10 mg group under fed conditions. In contrast, the overall mean E_max_ for the placebo group was 8.53%. The median time to E_max_ across all groups ranged from 2.75 to 6.00 h (Table 6).

**Table 6:** Summary of the E_max_ in cGMP from baseline and TE_max_

Dosage group	Participant number / statistic	E_max_ (%)	TE_max_(h)
**5 mg fasted**	S0102	-3.05	2.50
	S0103	-0.79	12.00
	S0104	21.82	6.00
	**Mean/median**	**5.99**	**6.00**
**7.5 mg fasted**	S0201	31.55*	6.00
	S0203	20.71	12.00
	S0204	41.37*	6.00
	**Mean/median**	**31.21**	**6.00**
**10 mg fasted**	S0301	20.25	8.00
	S0302	33.33*	2.50
	S0303	36.80*	3.00
	S0305	56.53*	2.50
	S0307	18.73	1.50
	S0308	-2.53	5.00
	**Mean/median**	**27.19**	**2.75**
**10 mg fed**	S0301	43.03*	1.50
	S0302	62.52*	3.00
	S0303	4.88	6.00
	S0305	39.73*	2.50
	S0307	37.23*	1.50
	S0308	22.24	3.00
	**Mean/median**	**34.94**	**2.75**
**Placebo**	S0101-fasted	26.55	6.00
	S0202-fasted	-3.05	0.50
	S0304-fasted	12.68	6.00
	S0306-fasted	15.32	8.00
	S0304-fed	8.01	3.00
	S0306-fed	-8.29	3.00
	**Mean/median**	**8.53**	**4.50**

Note: Mean for E_max_ and median for TE_max_

*Values greater than the max E_max_ of the placebo (26.55%)

**Fig. 5:**
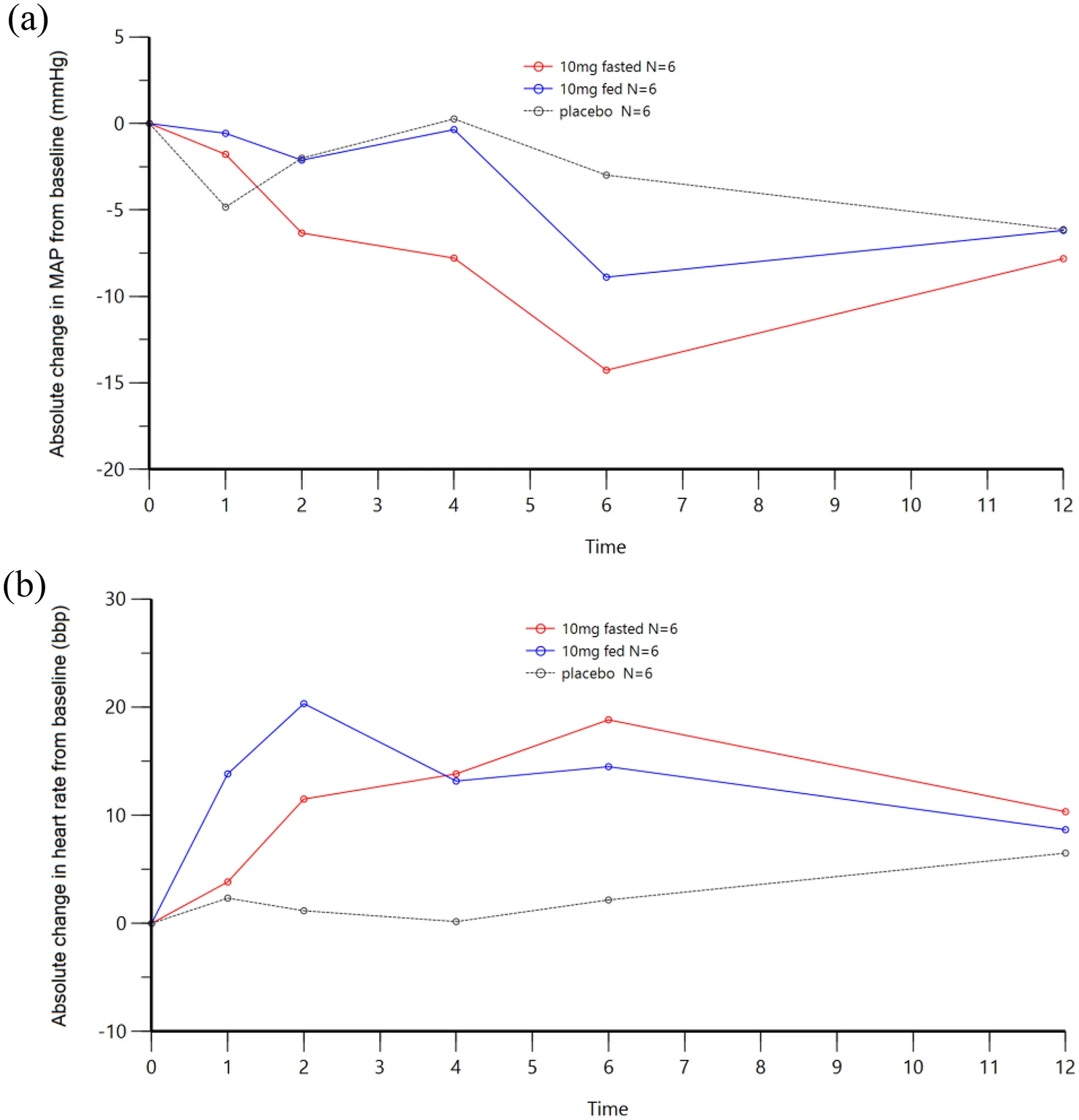
Absolute change in mean arterial pressure and heart rate for SK-08 10 mg. (**a**) Absolute change in mean arterial pressure versus baseline; (**b**) absolute change in heart rate versus baseline

Administration of SK-08 resulted in a blood pressure reduction, with the maximal change occurring at 4–6 h and returning to baseline by 12 h. A reflexive increase in heart rate was noted, reaching its peak at 6 h. The 10 mg group showed a more pronounced response effect (Fig. 5).

After a single oral dose of 10 mg SK-08 under fasting and high-fat meal conditions, the trends in changes of plasma cGMP, blood pressure, and heart rate were consistent, with no significant differences observed.

### Exposure-response relationship

**Fig. 6:**
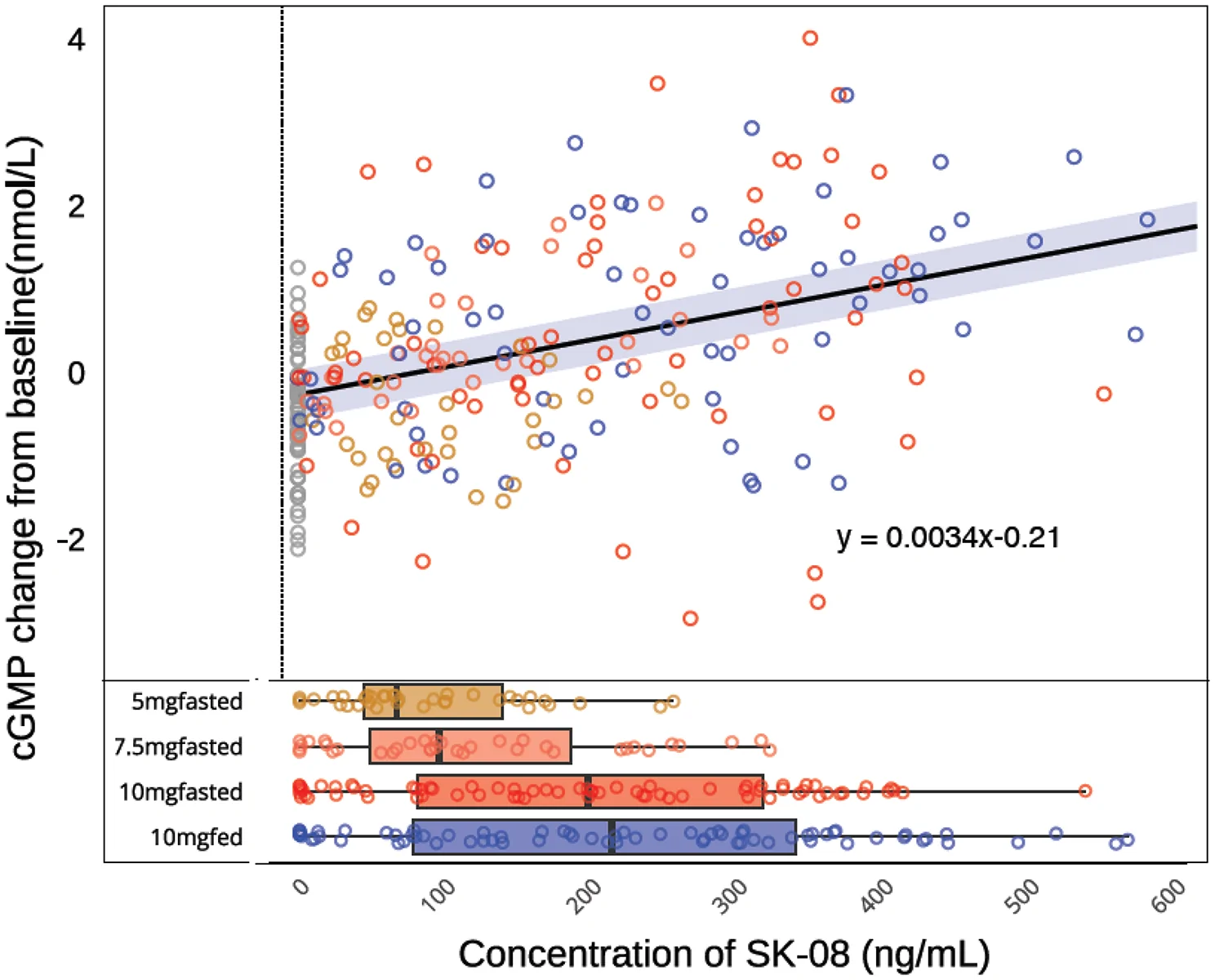
Exposure-response analysis between SK-08 concentration and cGMP change from baseline. Note: Open circles: individual observations; black line: linear regression (y=0.0034x−0.21); blue band: 90% CI. Boxplots: (median, IQR, 1.5×IQR whiskers). CI, confidence interval; IQR, inter-quartile range

To explore the exposure-response (E-R) relationship, cGMP-change from baseline was employed as an efficacy-related pharmacodynamic biomarker, while SK-08 plasma concentration served as the independent variable. Linear regression analyses demonstrated a concentration-dependent increase in cGMP change from baseline with rising SK-08 concentrations (Fig. 6). Notably, linear fitting of cGMP values against concentrations yielded a weak correlation (R²=0.35, without placebo). Further in-depth investigation of the E-R relationship will be conducted in patient cohorts via population modeling, as additional data are required.

**Fig. 7:**
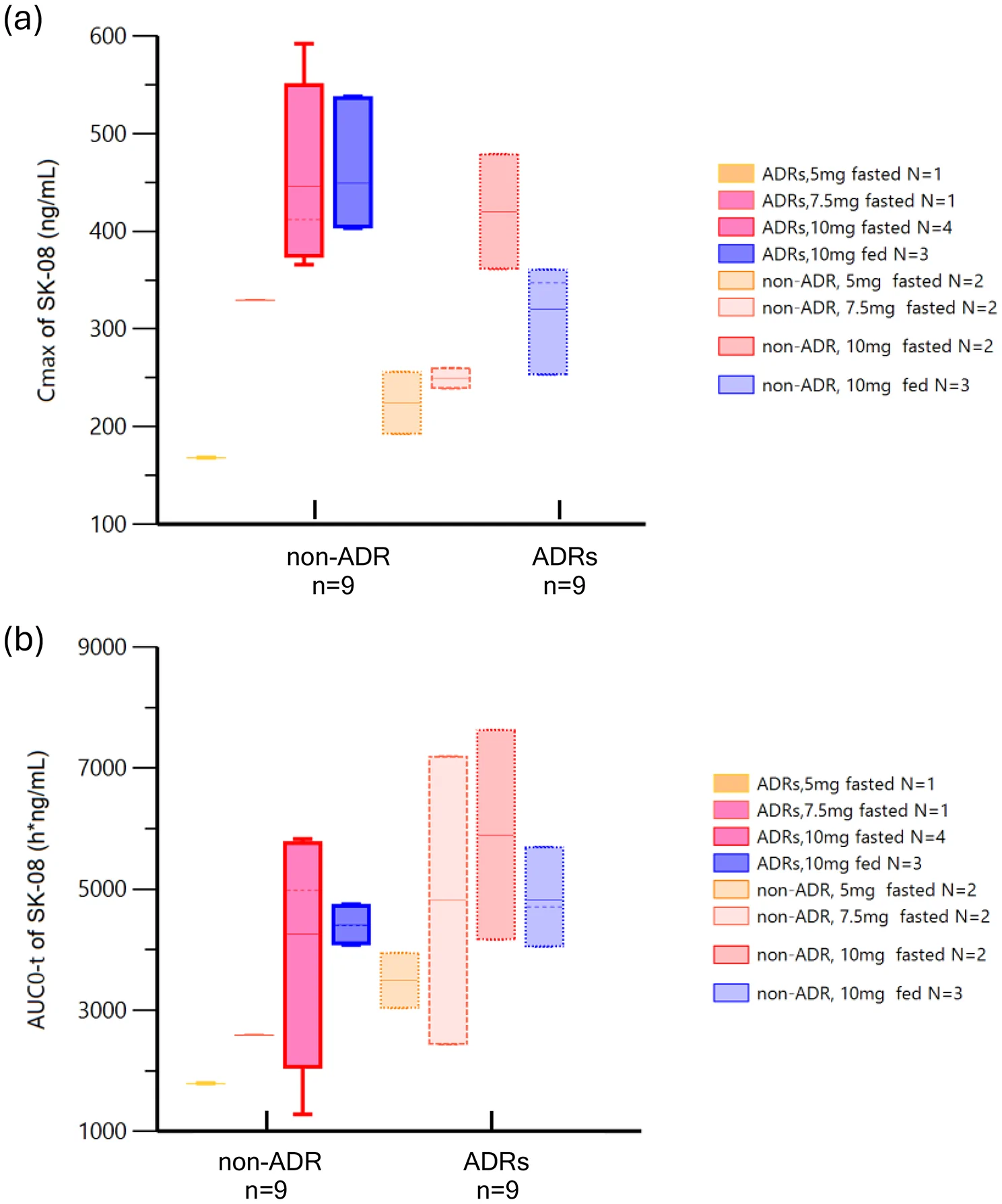
Exposure-response analysis between SK-08 and adverse drug reactions (ADRs). Note: (**a**) Cmax and (**b**) AUC0-t. Box plot details: median (solid line), arithmetic mean (dashed line); IQR (Q1–Q3, middle 50% of data); 1.5×IQR whiskers. IQR, inter-quartile range

Similarly, in a safety E-R relationship assessment, ADRs were assessed for an association with the C_max_ of SK-08. Analysis of all SK-08 dose groups stratified by the presence or absence of ADRs demonstrated that participants with a higher ADR incidence had correspondingly higher C_max_ (Fig. 7). However, this observation requires confirmation across a larger dose range and in a larger number of participants.

## Discussion

The results of this phase 1 study of oral SK-08, a novel sGC activator, in healthy participants verified the mechanism of action (MoA) and preliminarily demonstrated that SK-08 5, 7.5, and 10 mg have acceptable safety profiles and are well tolerated. All reported ADRs were mild to moderate in severity, with no SAEs, AEs leading to treatment discontinuation, or deaths. Hypotension (33.3%) and dizziness (16.7%) were the most frequently reported AEs, with all cases resolving without treatment with additional medications. Studies of other sGC modulators have demonstrated a similar safety profile to the present study. Treatment with vericiguat resulted in hypotension and syncope in one study [16], and an incidence of treatment-emergent AEs of 33.3–66.7% in another [17]. The most common AEs following treatment with riociguat are reported to be headache, gastritis, dizziness, nausea, diarrhea, hypotension, and vomiting [18]. Similarly, in a phase 1 trial of HEC95468, healthy participants reported mild headaches, dizziness, decreased blood pressure, and increased heart rate [19]. Therefore, SK-08 appears to have a similar safety profile to other sGC modulators, with no unexpected AEs. It should be noted that a relatively high proportion of participants in the placebo group experienced at least one AE (75%), which may be due to the small sample size in this group (*N* = 4), particularly as treatment assignment and AE evaluation was blinded.

The C_max_ and AUC_0−t_ in the SK-08 10 mg group were approximately twice those observed in the 5 mg group; whilst for the 7.5 mg group, no proportional relationship between exposure and dose was observed. However, due to the small sample size and inter-individual heterogeneity, further studies are required to investigate dose proportionality. Following a single oral dose of SK-08 7.5 mg or 10 mg, the mean maximum increase in cGMP reached approximately 30% above baseline in both groups, showing a notable pharmacological effect, suggesting that the systemic exposure of SK-08 at these doses are within the therapeutic range and there may be minimal delay between drug concentration and pharmacodynamic effect. However, the increase in cGMP in the 10 mg group was slightly lower than that in the 7.5 mg group. This may be due to the plasma drug concentration following the 10 mg dose quickly reaching a peak level and subsequently being rapidly eliminated.

An sGC modulator with a sharp absorption peak poses a risk to safety (a high incidence of hypotension), although stimulating an increase in cGMP requires a high plasma drug concentration [20, 21]. The SK-08 tablets utilized in this trial were immediate-release formulations with a fast peak and rapid elimination. Therefore, to reduce the risk of hypotension, strategies to soften the absorption peak of sGC modulators are required. One potential approach would be administration of the immediate-release formulation after meals to reduce speed of absorption. However, this would still require multiple daily administrations, which may negatively affect medication adherence. Alternatively, developing a controlled-release formulation, with a gradual increase in drug concentration and precise calibration of both the release rate and the duration of action, may allow maintenance of a relatively high plasma drug concentration level within the therapeutic range. This could support sustained cGMP production for extended efficacy and reduce adverse effects, such as hypotension, linked to rapid drug absorption. This approach is also consistent with the PK characteristics of drugs for this target (e.g., vericiguat, which has a relatively low peak-to-trough ratio [C_max_/C_min_≈2] to mitigate the risk of blood pressure fluctuation).

The safety profile and cGMP levels following fed administration were slightly better than those observed after administering an equivalent dose under fasted conditions. For example, all cases of hypotension in the 10 mg group occurred following fasted administration. This is possibly related to increased drug tolerance in participants after the second dose, but it requires further clinical trials for confirmation.

The increase in cGMP level following administration of SK-08 observed in this study suggests its potential as a novel treatment for CKD. This is evidenced by the strong cardiovascular efficacy of riociguat [22] and vericiguat [23], and the benefits in CKD demonstrated by avenciguat, an sGC activator [24]. These classes of agents exert their effects by elevating cGMP levels. Therefore, SK-08 has the potential to exert cardiovascular and renal protective effects based on the MoA demonstrated in this study. However, it should be noted that patients with CKD may have pathophysiological differences compared with the healthy participants of the present study such as altered NO–sGC signaling and hemodynamic sensitivity [25], and this limits the generalizability of the results of this study.

Despite limitations from the narrow dose range and limited sample size utilized in this trial, we were able to generate preliminary data to support a future investigation of a modified controlled-release formulation of SK-08. Specifically, the data provide preliminary evidence of a dose–effect relationship and plasma concentration fluctuation range of SK-08—both of which are critical for optimizing controlled-release formulations. Furthermore, leveraging experience from other sGC-based drugs, this trial was well-designed and rigorously controlled to ensure the safety of participants by avoiding exposure to unnecessarily high drug concentrations.

This study has several potential limitations. Firstly, as a single dose study in healthy volunteers, the PK, PD, and safety data may not be generalizable to patients with CKD, and the effect of comorbidities on the PK and safety of SK-08 were not assessed. Secondly, there was a sex imbalance between the dose groups, with no males enrolled into the 7.5 mg group. This limits the generalizability of the results, as sex can potentially impact PK and PD profiles. Finally, only 12 participants received the investigational drug, thus further studies with larger sample sizes are warranted.

## Conclusion

In summary, in healthy participants, SK-08 5–10 mg was well tolerated, with a safety profile comparable to that of similar sGC modulators. For the 10 mg dose, high fat food intake resulted in a mild PK effect (a slight T_max_ advance and ~ 10% reduction in C_max_/AUC) but did not appear to impact PD markers (cGMP, blood pressure, heart rate) or safety, although the incidence of AEs was lower compared with administration in a fasted state. Exposure-response analysis indicated a potential positive association between C_max_ and ADRs. However, maintaining a certain high drug concentration is required for efficacy, supporting the development of a controlled-release formulation to optimize the benefit-risk profile.

## Electronic Supplementary Material

Below is the link to the electronic supplementary material.

Supplementary Material 1

## Data Availability

Qualified researchers may request access to patient level data and related study documents including the clinical study report, study protocol with any amendments, blank case report form, statistical analysis plan, and dataset specifications. Patient level data will be anonymised, and study documents will be redacted to protect the privacy of our trial participants. Further details on data sharing criteria, eligible studies, and process for requesting access can be found at: http://www.clinicalstudydatarequest.com/.

## Declarations

### Patient and public involvement

Patients and public were not involved in the in the design, conduct and reporting of the trial.

## Abbreviations

ADR: Adverse drug reaction
AE: Adverse event
AUC_0−t_: Area under the concentration time curve from 0 to the time of the last quantifiable concentration
AUC_0–∞_: Area under the concentration time curve from 0 to infinity
AUC__%Extrap_: The percentage of AUC due to extrapolation beyond the last measurable concentration
cGMP: cyclic guanosine 3’, 5’-monophosphate
CI: Confidence interval
CKD: Chronic kidney disease
CL/F: Apparent clearance
C_max_: Maximum plasma concentration
CTCAE: Criteria for adverse events
ECG: Electrocardiogram
E_max_: Maximum percentage change
MoA: Mechanism of action
NO: Nitric oxide
NOAEL: No-observed-adverse-effect level
PD: Pharmacodynamic
PK: Pharmacokinetic
SAE: Serious adverse event
SD: Standard deviation
sGC: soluble guanylate cyclase
T_1/2_: Elimination half-life
T_max_: Time to maximum plasma concentration
V_z_/F: Apparent volume of distribution adjusted for bioavailability
