# Supplementary material for: Safety, pharmacokinetic, and pharmacodynamic characteristics of SK-08, a soluble guanylate cyclase activator, after oral administration in healthy participants: a randomized phase 1 trial
Source: BMC Nephrol. 2026 Jul 9;27:586. doi: 10.1186/s12882-026-05138-y (PMC13640110; doi:10.1186/s12882-026-05138-y)
Supplement: Supplementary file 1 [file 12882_2026_5138_MOESM1_ESM.docx]

**Supplemental Appendix**

**Exclusion Criteria**

Participants who meet any of the following criteria shall not be enrolled in this trial:

1. Have a specific history of allergy (to food, drugs, etc.) or are allergic in constitution (such as known to be allergic to two or more substances), or have a known history of allergy to SK08 and its related excipients (lactose monohydrate, microcrystalline cellulose, croscarmellose sodium, hydroxypropyl methylcellulose (LV), hydrophobic colloidal silicon dioxide, magnesium stearate, titanium dioxide, sodium lauryl sulfate, talc, polyvinyl alcohol, caprylic/capric mono- and diglycerides) (by inquiry);
2. Have a history of chronic or serious diseases of the cardiovascular, hepatic, renal, biliary, respiratory, hematologic and lymphatic, endocrine, immune, psychiatric, neuromuscular, or gastrointestinal systems, within 1 year before screening and before randomization, or have a history of gastrointestinal disease (including existing conditions) within 1 year before screening and before randomization, including chronic or active upper gastrointestinal diseases such as esophageal disease, gastritis, duodenitis, peptic ulcer, or active gastrointestinal bleeding, or have undergone gastrointestinal surgery (by inquiry);
3. Have clinically significant abnormalities judged by the investigator in vital signs, physical examination, clinical laboratory tests (blood routine, urine routine, blood biochemistry, coagulation function, four transfusion-related tests), 12-lead ECG, abdominal B-ultrasound, or orthostatic hypotension test results at screening (by examination);
4. Have suffered from acute diseases from 2 weeks before screening to before randomization (by inquiry);
5. Have a history of organic heart disease, heart failure, myocardial infarction, angina pectoris, coronary artery bypass surgery, angioplasty, vascular stent implantation, uncontrolled hypotension, left ventricular ejection fraction below the lower limit of normal, unexplained arrhythmia, ventricular tachycardia, atrioventricular block, QT prolongation syndrome, or symptoms and family history of QT prolongation syndrome, and are judged by the investigator to be unsuitable for participation in this trial (by inquiry);
6. Have a history or presence of hypotension or hypovolemia, intracranial hypertension or cerebral hemorrhage, or have eye diseases (such as angle-closure glaucoma) and are assessed by the investigator as unsuitable for inclusion (by inquiry);
7. Participants with a past or present history of syncope or frequent dizziness (≥2 episodes within the past 6 months) (by inquiry);
8. Participants who have started a significantly abnormal diet (such as high-potassium, low-fat, fasting, or low-sodium diets) within 30 days prior to screening (by inquiry);
9. Participants with hypokalemia, hyperkalemia, hypomagnesemia, hypermagnesemia, hypocalcemia, or hypercalcemia that are considered clinically significant by the investigator (by examination);
10. Participants with any of the following 12-lead ECG findings at screening, judged as clinically significant by the investigator:

1) PR ≥ 200 ms;

2) QTcF ≥ 440 ms;

3) QRS ≥ 100 ms;

4) QT ≥ 500 ms (by examination).

11. Participants who have used any medication (including prescription drugs, over-the-counter drugs, traditional Chinese medicine, or health supplements) within 2 weeks prior to screening and before randomization (by inquiry);
12. Participants who have used, within 30 days prior to screening and before randomization, any drug that may interact with the investigational product, such as CYP450 inhibitors or inducers (e.g., barbiturates, carbamazepine, ketoconazole, itraconazole, etc.) (by inquiry);
13. Participants who have consumed, within 48 hours before dosing, caffeine- or alcohol-containing beverages, or foods affecting drug metabolism such as chocolate, dragon fruit, mango, starfruit, guava, pomelo, orange, grapefruit, or grapefruit products (by inquiry);
14. Participants who have undergone major surgery (excluding diagnostic surgery) within 6 months prior to screening and before randomization, or who plan to undergo surgery during the study period, or who have undergone any surgery that may affect drug absorption, distribution, metabolism, or excretion as judged by the investigator (by inquiry);
15. Participants who have received a live attenuated vaccine within 2 weeks prior to screening and before randomization, or who will require such vaccination during the study period (by inquiry);
16. Participants who have not completed participation in another drug or device clinical trial within 3 months prior to screening, where completion is defined as the date of last study withdrawal (by inquiry and verification);
17. Participants who have donated blood within 3 months prior to screening, or have had total blood loss ≥400 mL (excluding menstrual blood loss) due to donation or other causes within the past 6 months, or plan to donate blood within 3 months after the last dose (or within 6 months for a food-effect study group) (by inquiry);
18. Participants with a history of alcohol abuse within 1 year prior to screening, defined as an average weekly intake exceeding 14 units of alcohol (1 unit = 360 mL beer or 45 mL 40% spirits or 150 mL wine), or those unable to abstain from alcohol during the study, or with a positive alcohol breath test (>0.0 mg/100 mL) (by inquiry and examination);
19. Participants who have smoked more than 5 cigarettes per day within 3 months prior to screening and before randomization, or who cannot abstain from any tobacco products during the study (by inquiry);
20. Participants with a history of drug abuse (including non-medical use of any narcotic or psychotropic substances) within 1 year prior to screening and before randomization, or those with positive results on drug screening (including morphine, methamphetamine, ketamine, MDMA (ecstasy), or cannabis (THC acid), etc.) (by inquiry and examination);
21. Participants unable to tolerate venipuncture or indwelling needles, or with a history of syncope or fainting at the sight of needles or blood (by inquiry);
22. Participants with lactose intolerance (by inquiry);
23. Participants with special dietary requirements who cannot comply with a standardized diet (by inquiry);
24. Participants with difficulty in swallowing (by inquiry);
25. Any other Participants deemed unsuitable for participation by the investigator.
26. In addition to the above criteria, female Participants shall also be excluded if they meet any of the following conditions:
27. Use of oral contraceptives within 30 days prior to screening and before randomization (by inquiry);
28. Use of long-acting estrogen or progestin injections (including progestin-containing intrauterine devices or implants) within 6 months prior to screening and before randomization (by inquiry);
29. Having engaged in unprotected sexual intercourse with a partner within 14 days prior to screening and before randomization (by inquiry);

30. Positive pregnancy test (by examination);

31. Pregnant or breastfeeding women (by inquiry).

**Meal composition**

A high-fat meal with a total calorie intake of less than 800 kcal, or a high-fat meal containing less than 50 kcal of calories from fat.

| **High-fat-calorie meal plan** | | | | | |
| --- | --- | --- | --- | --- | --- |
| Food item | Quantity | Composition | Nutrient content | | |
|  |  |  | Protein (g) | Fat (g) | Carbohydrate (g) |
| Whole milk | 250 mL | 250 mL | 9 | 10.5 | 12.5 |
|  |  | Energy (kcal) | 36 | 94.5 | 50 |
| Fried eggs | 2 | Eggs 100 g | 14 | 8.8 | 2.8 |
|  |  | Energy (kcal) | 56 | 79.2 | 11.2 |
|  |  | Cooking oil 30 g |  | 30 |  |
|  |  | Energy (kcal) | 0 | 270 | 0 |
| Pancake | 1 | Wheat Flour 60 g | 4.8 | 0.88 | 44 |
|  |  | Energy (kcal) | 19.2 | 7.92 | 176 |
|  |  | Lean meat 50 g | 10 | 3 | 0.7 |
|  |  | Energy (kcal) | 40 | 27 | 2.8 |
|  |  | Cooking oil 10 g |  | 10 |  |
|  |  | Energy (kcal) | 0 | 90 | 0 |
| Total |  | Nutrients (g) | 37.8 | 63.18 | 60 |
| Total |  | Calories (kcal) | 151.2 | 568.62 | 240 |
| Total |  | Total energy (kcal) |  | 959.82 |  |
